# Identification and Expression Analysis of the *C-TERMINALLY ENCODED PEPTIDE* Family in *Pisum sativum* L.

**DOI:** 10.3390/ijms232314875

**Published:** 2022-11-28

**Authors:** Maria A. Lebedeva, Maria S. Gancheva, Olga A. Kulaeva, Evgeny A. Zorin, Daria A. Dobychkina, Daria A. Romanyuk, Anton S. Sulima, Vladimir A. Zhukov, Lyudmila A. Lutova

**Affiliations:** 1 Department of Genetics and Biotechnology, Saint Petersburg State University, Universitetskaya emb.7/9, Saint Petersburg 199034, Russia; 2 Laboratory of Genetics of Plant-Microbe Interactions, All-Russia Research Institute for Agricultural Microbiology (ARRIAM), Podbelsky Sh. 3, Saint Petersburg 196608, Russia

**Keywords:** CEP, nitrate, nitrogen-fixing nodules

## Abstract

The *C-TERMINALLY ENCODED PEPTIDE*(CEP) peptides play crucial roles in plant growth and response to environmental factors. These peptides were characterized as positive regulators of symbiotic nodule development in legume plants. However, little is known about the CEP peptide family in pea. Here, we discovered in pea genome 21 *CEP* genes (*PsCEPs*), among which three genes contained additional conserved motifs corresponding to the PIP (PAMP-induced secreted peptides) consensus sequences. We characterized the expression patterns of pea *PsCEP* genes based on transcriptomic data, and for six *PsCEP* genes with high expression levels in the root and symbiotic nodules the detailed expression analysis at different stages of symbiosis and in response to nitrate treatment was performed. We suggest that at least three *PsCEP* genes, *PsCEP1, PsCEP7* and *PsCEP2*, could play a role in symbiotic nodule development, whereas the *PsCEP1* and *PsCEP13* genes, downregulated by nitrate addition, could be involved in regulation of nitrate-dependent processes in pea. Further functional studies are required to elucidate the functions of these *PsCEP* genes.

## 1. Introduction

In order to survive in a changing environment, plants have to adapt their developmental processes to diverse abiotic and biotic factors. Recent studies have highlighted the emerging role of peptide hormones in the systemic coordination of plant growth in response to environmental factors. Among them, the CEP (*C-TERMINALLY ENCODED PEPTIDE*) peptides were characterized as essential regulators of root and symbiotic nodule development [1]. The CEPs represent a group of post-translationally modified peptide hormones found in seed plants. The mature CEP peptides consist of 15 amino acids and are produced from the conserved CEP domain of a precursor protein due to proteolysis and post-translationally modifications [2,3,4]. Specifically, in CEP peptides, the conserved proline residues are hydroxylated and some of them are arabinosylated, and such modifications are supposed to be important for their receptor recognition and biological activity, as shown for other groups of post-translationally modified peptides [4,5,6].

In *Arabidopsis thaliana*, the *CEP* genes are activated in response to nutrient deficiency and other stress factors and function as negative regulators of root system development [2]. The receptors of CEP peptides are LRR-RLK (CEPR1 и CEPR2 (CEP RECEPTOR) in *A. thaliana*, which were shown to act in the shoot and mediate a systemic effect on nitrogen deficiency. Specifically, in split-root system experiments with heterogenic nitrogen content in the growth media, activation of CEPRs by CEP peptides derived the roots grown under nitrogen deficiency activated the long-distance signaling pathway to upregulate the expression of nitrate transporter genes in the roots growing under high nitrogen conditions [7]. Moreover, the CEP peptides were shown to inhibit lateral root development through a local mechanism [8,9,10].

In addition to their role in lateral root development, the CEP peptides were shown to positively regulate the development of nitrogen-fixing nodules formed on the roots of legume plants due to symbiotic interaction with soil bacteria rhizobia [3,9,11,12]. Symbiotic nodulation could be regarded as a response of a host plant to nitrogen deficiency, which allows the legume plants to get available nitrogen from the symbiotic nitrogen fixation. At the same time, a high amount of nitrogen in the growth media inhibits nodulation [13]. In *Medicago truncatula*, the MtCEP1 peptide enhanced symbiotic nodule development in a systemic manner through a shoot-acting receptor, encoded by the *MtCRA2 (COMPACT ROOT SYSTEM ARCHITECTURE 2)* gene. In contrast, the MtCEP1 peptide inhibited lateral root development locally through a root-acting MtCRA2 receptor [10]. Moreover, it was found that activation of shoot-acting MtCRA2 receptor protein by the root-derived CEP peptides resulted in accumulation of microRNA miR2111 in the shoot. This microRNA acts as a shoot-to root transported systemic regulator and targets the transcripts of *TOO MUCH LOVE* (*TML*) genes (which encode negative regulators of symbiosis) in the root, thereby enhancing host plant competence to nodulation [14]. In addition to *MtCEP1*, the *MtCEP7* gene positively regulates nodulation: its constitutive expression systemically increased nodule number in roots through the activity of the MtCRA2 receptor in shoots [11]. Moreover, the *MtCEP7* gene is directly activated by the NIN (NODULE INCEPTION) transcription factor, which acts as a master regulator of legume-rhizobia symbiosis [11]. Recently, *MtCEP1* was shown to be downregulated by the nitrate via the MtNLP1 (NIN LIKE PROTEIN 1) transcription factor, which binds to the *MtCEP1* promoter, mediating nitrate-dependent inhibition of its expression [15]. Furthermore, multigene editing revealed that *MtCEP1/2/12* redundantly control lateral root and nodule number in *M. truncatula* [12]. Triple *Mtcep* mutants obtained by CRISPR/Cas9-mediated gene-editing had a significantly lower number of symbiotic nodules, whereas lateral root density was higher in mutant plants [12]. Therefore, at least four *CEP* genes in *M. truncatula, MtCEP1, MtCEP2, MtCEP7,* and *MtCEP12,* are involved in symbiotic nodule development. Interestingly, the CEP peptides derived from the *MtCEP1* and *MtCEP2* genes were found in the xylem sap of *M. truncatula* [16]. Moreover, the CEP peptide homologous to MtCEP12 was found in the xylem sap of soybean [17,18]. Therefore, these CEP peptides could mediate systemic effects in plant development.

To further expand our understanding of CEP peptides in plant development and symbiotic nodulation, we performed a comprehensive identification of the CEP peptide family in pea. This group of peptides has not been studied in pea earlier. Moreover, we analyzed the expression patterns of *PsCEP* genes based on available transcriptomic data and by qPCR analysis with a special focus on symbiotic nodulation.

## 2. Results

### 2.1. Identification of the CEP Peptide Family in Pea Genome

To identify the *PsCEP* genes, the full-length sequences of the *CEP* genes from *Medicago truncatula* [19] and *Arabidopsis thaliana* were used as queries to perform BLASTn against genomic (https://urgi.versailles.inra.fr/download/pea/, accessed on 5 July 2022) [20] and transcriptomic data [21] of pea cv. Cameor. As a result, 21 sequences encoding CEP peptides were identified. The corresponding proteins ranged in size from 84 up to 134 amino acids. The nucleotide sequences of the *PsCEP* genes and corresponding amino acid sequences are presented in Supplementary Table S1.

**Figure 1:**
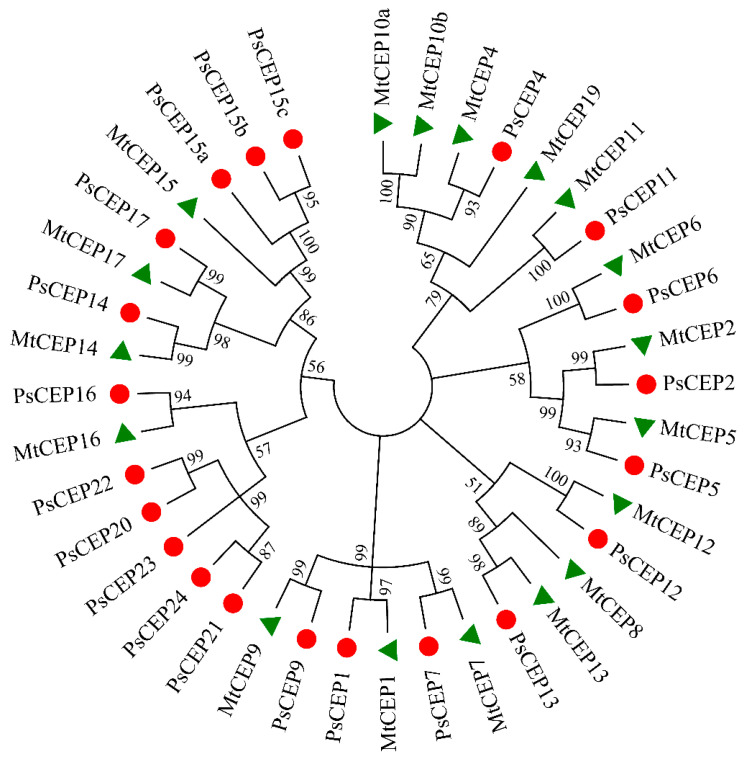
Phylogenetic tree built based on amino acid sequences of the PsCEP and MtCEP proteins. PsCEPs are marked with red circles, MtCEPs are marked with green triangles.

Phylogenetic analysis revealed the relationship between the identified PsCEP sequences and their homologues in *M. truncatula* (Figure 1).

The closest homologous counterparts for the *MtCEPs* genes identified by Boschiero et al. were found in pea, except for the *MtCEP8, MtCEP10,* and *MtCEP19* genes. The corresponding *PsCEP* genes were designated in accordance with their closest homologues found in *M. truncatula* (*PsCEP1, PsCEP2, PsCEP4-7, PsCEP9, PsCEP11-17*) to simplify further discussions of their functions in two species. The *MtCEP3* gene was initially identified in the study by Imin et al. (see Figure S1 in [3]), but the corresponding gene was absent in the latest versions of *M. truncatula* genome assembly according to Boschiero et al., 2020 [19]. Similarly, we failed to find sequences with high similarity to *MtCEP3* in pea genome assemblies. The *MtCEP19* gene was identified by Boschiero et al. However, its sequence was not found in the MtrunA17r5.0-ANR genome assembly, and sequences with high similarity to *MtCEP19* was absent in pea genome. Interestingly, three *PsCEPs* genes with high sequence similarity to the *MtCEP15* gene were identified, which were named as *PsCEP15a-c*. In addition to this, five putative precursors of *PsCEPs* clustered together in one group were identified, which were designated as *PsCEP20-24* (Figure 1). These genes form a separate branch of the *PsCEP* genes and do not have close homologues among the annotated *MtCEP* genes.

### 2.2. Chromosome Localization of the PsCEP Genes

**Figure 2:**
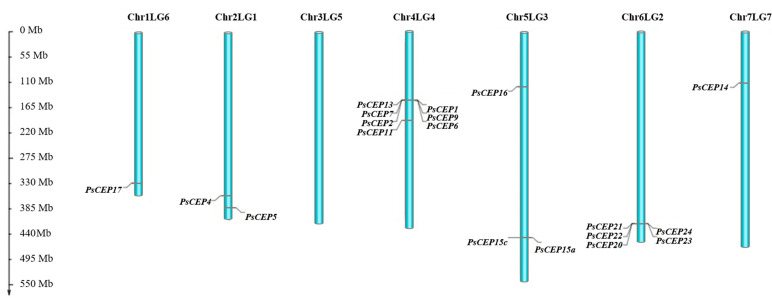
Chromosome localization of the *PsCEP* genes according to the INRA version of pea genome assembly. The positions of the *PsCEP12* and *PsCEP15b* genes are not shown since the chromosome localization of these genes was not determined in the INRA version of pea genome assembly [20].

**Figure 3:**
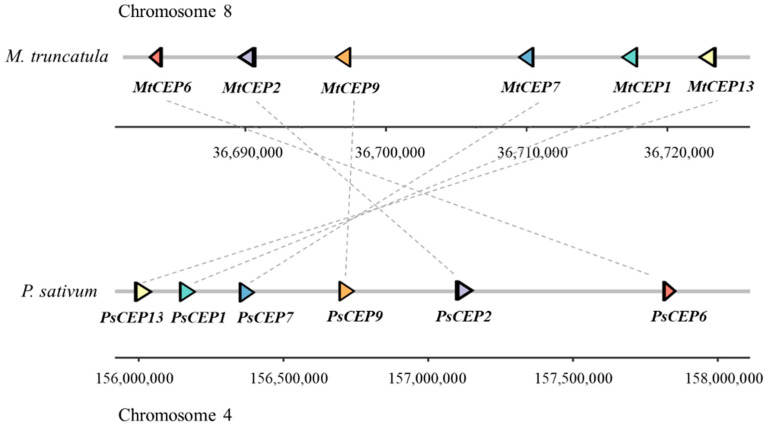
Microsynteny of the genomic regions bearing the cluster of the *CEP* genes in *M. truncatula* and *P. sativum*. The positions of the orthologous genes are shown with the triangles of the same color and are connected with dotted lines.

Next, we analyzed chromosome localization of the *PsCEP* genes (Figure 2). The chromosome 3 (Chr3LG5) lacks *PsCEPs*, whereas chromosomes 1 (Chr1LG6) and 7 (Chr7LG7) contained only one *PsCEP* gene, *PsCEP17* and *PsCEP14*, respectively. Other chromosomes bear several *PsCEPs*, and some of them were organized in a cluster. For example, the *PsCEP13*, *PsCEP1*, *PsCEP7*, *PsCEP9*, *PsCEP2*, and *PsCEP6* are located as a cluster on chromosome 4 (Chr4LG4) (Figure 2 and Figure 3).

A microsynteny analysis revealed that the closest homologs of these genes are located in a syntenic region on chromosome 8 of *M. truncatula*, suggesting that corresponding genes in both legumes are orthologous to each other (Figure 3).

Interestingly, the *PsCEP20-24* genes, which form a single branch according to phylogenetic analysis, are located on chromosome 6 (Chr6LG2) next to each other (Figure 2).

### 2.3. The Structural Elements of the PsCEP Proteins

**Figure 4:**
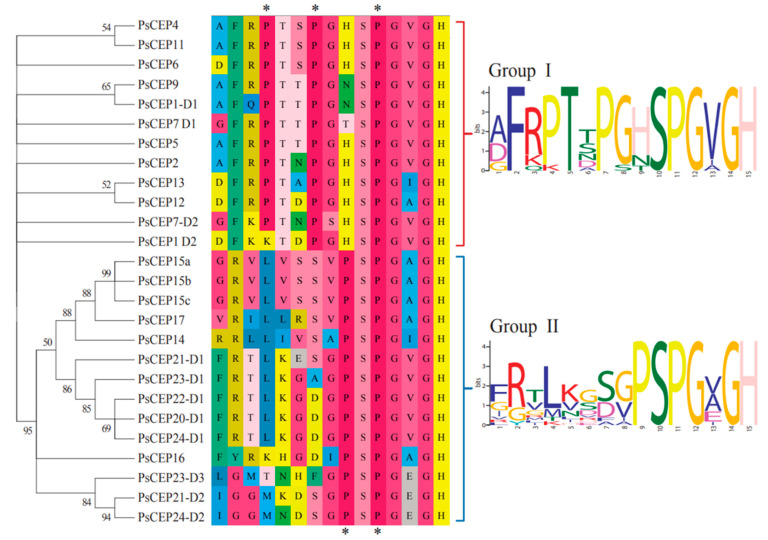
Alignment of the PsCEP peptide sequences. WebLogo representation of the CEP domains from two groups is shown. Asterisks mark the conserved Pro residues found in two groups of the CEP domains.

Next, we analyzed the structural elements of the PsCEP proteins, including the CEP motifs, the N-terminal signal peptide, and putative cleavage site (Supplementary Tables S2 and S3). MEME and FIMO tools were used for the prediction of domains in PsCEP protein sequences, and the results were checked by manual verification. PsCEP1 and PsCEP7 contain two CEP domains (designated as D1 and D2). The precursor proteins encoded by *PsCEP20*-*PsCEP24* genes also contain two or three conserved domains (D1–D3). Based on the amino acid sequences, the CEP domains were clustered into two subgroups, group I (CEP-I) and group II (CEP-II) (Figure 4). All of them contain the conserved Pro11 residue. The representatives of group I (except for D2 of PsCEP1) have the conserved Pro4 residue, as well as the conserved Pro7. In contrast to the group I CEP peptides, the group II CEP peptides have a conserved Pro9 residue, but lack Pro4 and Pro7. Therefore, these two groups have the common conserved Pro11 residue, but differ in conserved Pro residues in other positions.

**Figure 5:**
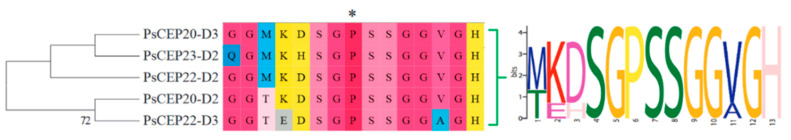
Alignment of the PIP peptide sequences found in the PsCEP20, PsCEP22 and PsCEP23 precursor proteins. WebLogo representation of the conserved domain is shown. The Pro8 residue is marked with asterisk.

Interestingly, we found one more type of a conserved domain encoded by the *PsCEP* genes from the *PsCEP20*-*24* branch. In addition to the CEP-II domain, the PsCEP20, PsCEP22, and PsCEP23 proteins have a conserved domain that does not match the CEP domain consensus and contains SGPSSGG sequence with the conserved Pro8 residue lacking the Pro residues in other positions (Figure 5). FIMO search in *M. truncatula* and *A. thaliana* genomes revealed that this domain matches the consensus sequences of the PIP-like (PAMP-induced secreted peptides) peptides, which represent a distinct group of regulatory peptides characterized in plants (Hou et al., 2015). PsCEP20 and PsCEP22 contain two PIP-like domains, whereas PsCEP23 contains one PIP-like motif in addition to the CEP domains (Supplementary Table S2). The closest homologue of the genes from the *PsCEP20*-*24* branch genes in *M. truncatula* is the *MtPIP8* gene. The MtPIP8 precursor protein also contains two different conserved domains, the D1 domain corresponding to the CEP peptide and the D2 domain corresponding to the PIP peptide (Supplementary Figure S1). Two other proteins from this group, PsCEP21 and PsCEP24, contain two CEP domains and lack the PIP-like domains. These findings hint at the possible common evolutionary origin of the CEP and PIP peptides.

Moreover, to analyze possible duplication events during the evolution of the *PsCEP* genes, a HSDFinder tool [22] was used. We found pairs of the PsCEP proteins with a high degree of similarity in both pairwise sequence identity (over 80%) and sequence length (less than 10% of amino acid length variance). The first group of proteins with a high degree of similarity included PsCEP15a, PsCEP15b, and PsCEP15c. In the second group, PsCEP20 and PsCEP22 showed a high degree of similarity, whereas in the third group PsCEP21 and PsCEP24 showed a high degree of similarity. Therefore, the genes within these groups might have evolved due duplication events. At the same time, the analysis of the sequence similarity of the of the PsCEP peptides and the chromosome localization of the *PsCEP* genes using MCScanX [23] did not allow us to identify certain collinear blocks. Apparently, this may be due to the distributed arrangement of the *PsCEP* genes on chromosomes.

**Figure 6:**
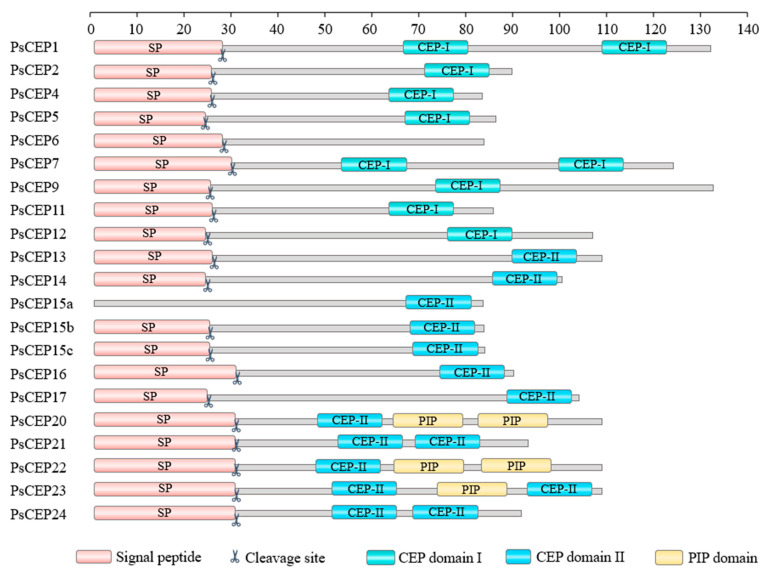
Structural organization of the PsCEP proteins. The conserved functional domains (CEP-I, CEP-II, PIP), N-terminal signal peptide (SP) and putative cleavage site (CS) are shown.

In addition to the conserved functional domains, we analyzed the positions of N-terminal signal peptides and putative cleavage site in the PsCEP precursor proteins. All PsCEPs appeared to have signal peptides and putative cleavage site revealed by the Signal IP ver.6.0 online service except for the *PsCEP15a* gene (Supplementary Table S3, Figure 6). PsCEP15a lacks Leu residues in the very N-terminal part of the protein, and its 3–12 positions differ from those of the PsCEP15b and PsCEP15c proteins. However, the upstream residues of these proteins are almost identical. Therefore, the *PsCEP15a* gene unlikely encodes a functional peptide due to the loss of the signal peptide domain.

### 2.4. Cis-Regulatory Elements in the Promoters of the PsCEP Genes

**Figure 7:**
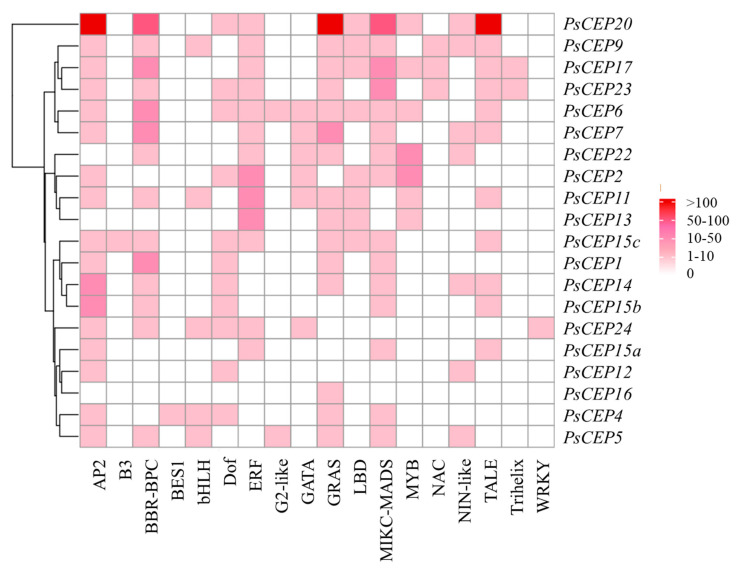
Cis–acting elements found in the promoters of the *PsCEP* genes. The color scale bar indicates the numbers of cis-acting elements in the promoters.

The members of the *CEP* gene family are known to be regulated by environmental cues [2]. To study possible regulators of the *PsCEPs* expression we searched for the conserved cis-regulatory elements in the promoter sequences of the *PsCEP* genes (see Supplementary Table S1) using the PlantRegMap platform [24]. The most frequently occurring cis-regulatory elements in the promoters of the *PsCEP* genes are the motifs targeted by AP2 (found in the promoters of 17 *PsCEP* genes), GRAS and MIKC-MADS (found in the promoters of 15 *PsCEP* genes), and BBR-BPC and ERF transcription factors (found in the promoters of 14 and 15 *PsCEP* genes, respectively) (Figure 7). Members of AP2/ERF family transcription factors were characterized as key regulators of various stress responses in plants, whereas GRAS, BBR-BPC, and MIKC-MADS transcription factors are known as important regulators of plant development. The prevalence of these regulatory elements in the promoters of *PsCEP* genes hints that PsCEPs could regulate plant growth and development in response to different stress factors. The binding sites for other transcriptional regulators of plant development, such as LBD, GATA, and TALE transcription factors, were also found in the promoters of the *PsCEP* genes, suggesting the involvement of PsCEPs in plant growth regulation.

**Figure 8:**
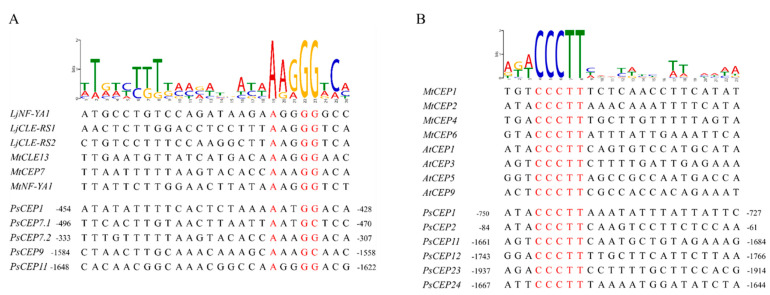
NIN-binding sites (according to Laffont et al. [11]) (**A**) and hNRE motifs (according to Luo et al. [15]) (**B**) identified in the promoters of the *PsCEP* genes.

Previously, it was shown that the *CEP* genes are upregulated in response to nitrogen starvation and symbiotic nodule development on the root [2,11,25]. Both these pathways are regulated by the NIN-like transcription factors: NIN acts as a key regulator of nodule development, whereas NLPs (NIN-like proteins) are responsible for nitrate-regulated gene expression [26]. In *M. truncatula*, the NIN transcription factors were reported to bind directly to the promoters of *MtCEP1* and *MtCEP7* genes, respectively [11,15]. Using PlantRegMap platform, we revealed the binding sites for NIN-like proteins in the promoters of several *PsCEP* genes. However, the consensus sequences for the NIN-binding sites (NBS) and NRE (nitrate-responsive elements) motifs, targeted by the NLP (NIN-like proteins) in response to nitrate, were revised in several recent studies [11,15,27,28]. It was shown that the NIN and NLP transcription factors have slight different DNA-binding specificities [28]. In *L. japonicus*, the features of the LjNIN-specific binding sites targeted by the NIN homodimer and binding sites for the LjNLP4/LjNIN heterodimers were characterized. They both consist of 30 nucleotides, in which there are two motifs with semi-palindromic structures, whereas LjNIN-specific binding sites had weaker palindromic structures in contrast to the LjNLP4/LjNIN common binding sites [28]. Therefore, we searched for the conserved binding sites for the NIN and NLP transcription factors in the promoters of the *PsCEP* genes using the motifs described in the study by Nishida et al., 2021 [28]. We found 14 putative NIN/NLP binding sites that could be targeted by the NIN-NLP heterodimers and 10 putative NIN binding sites that could be activated by the NIN transcription factor (Supplementary Figure S2). In the work by Laffont et al. [11], the *MtCEP7* gene was described as a target of the NIN transcription factor during symbiosis, and the NIN-binding consensus sequence was described in this study [11]. We searched for the motif described by Laffont et al. [11] in the promoters of *PsCEP* genes and found that the *PsCEP7* gene has two putative NIN-binding sites in its promoter, whereas the PsCEP1, *PsCEP9*, and *PsCEP11* genes have one putative NBS (Figure 8A). NBS (PsCEP7.2) in the promoter of *PsCEP7* gene identified using this consensus sequence is similar to the one identified as the NIN-specific target using the motif described by Nishida et al., 2021 [28] (Supplementary Figure S2). Moreover, recently, it was shown that MtNLP1 binds to the *MtCEP1* promoter through specific half NRE (hNRE) motifs to repress its expression in the presence of nitrate [15]. We found hNRE motifs described by Luo et al. in the promoters of six *PsCEP* genes (Figure 8B). Interestingly, the upstream regulatory sequences of the *PsCEP1* and *PsCEP2* genes contain the hNRE motifs similar to their closest homologues in *M. truncatula*. These genes could be downregulated by the NLP transcription factor in a similar way to that described for *MtCEP1* gene [15].

### 2.5. Expression Analysis of the PsCEP Genes Based on Transcriptomic Data

**Figure 9:**
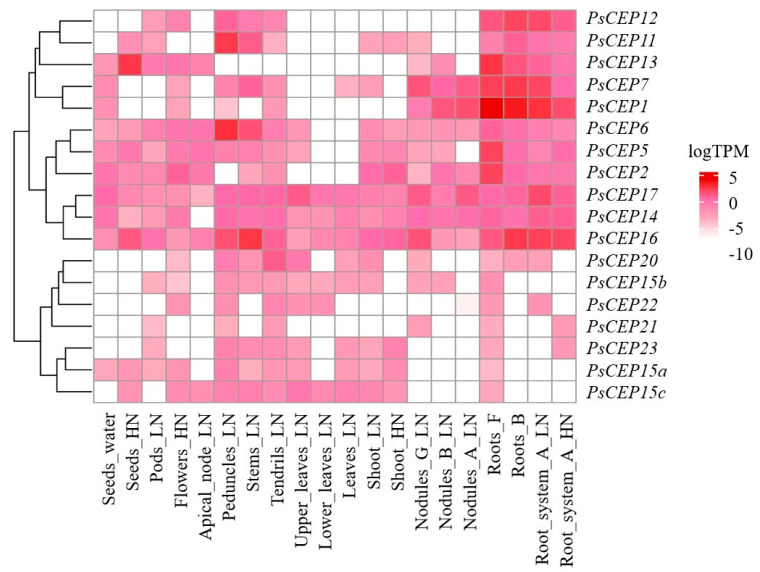
Heatmap illustrating the expression levels of the *PsCEP* genes in different tissues according to transcriptomic studies by Alves-Carvalho et al. [21]. Only those genes are shown, which have TPM values > 0. HN—high nitrate condition, LN—low nitrate condition, A, B, F, G—stages of pea development (A—7–8 nodes, 5–6 opened leaves; B—the start of flowering; F—8 days after sowing; G—18 days after sowing, i.e., 10 days after inoculation).

Next, we analyzed the expression patterns of identified *PsCEP*s in different organs using available transcriptomic data. First, we used transcriptomic data obtained by Alves-Carvalho et al., which encompass the transcript abundance (TPM) in different pea organs. According to these data, the transcripts of the *PsCEP1,2,5,7,12* genes demonstrate highest expression levels in the root system. *PsCEP6* and *PsCEP11* are more abundant in peduncles and stems. Transcripts of *PsCEP13* are more abundant in roots and seeds (under high nitrate condition), whereas *PsCEP16* demonstrates relatively high abundance in seeds (under high nitrate condition), peduncles, stems, nodules, and roots (Figure 9). The *PsCEP1, -7, -16,* and -*17* genes are abundant in symbiotic nodules, suggesting these genes could be involved in nodule development. Finally, the transcripts of *PsCEP20-23* and *PsCEP15a-c* have lower expression levels and do not demonstrate high abundance in the root system.

**Figure 10:**
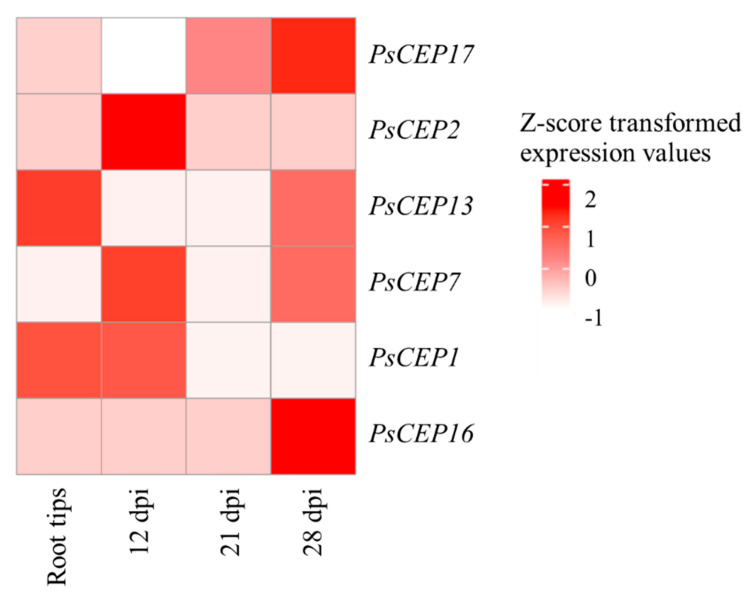
Heatmap illustrating the expression levels of the *PsCEP* genes in symbiotic nodules. Only those *PsCEP* genes which have transcript abundance ≥10 according to the data by Zorin et al. [29] are shown.

To study the expression levels of the *PsCEPs* genes in symbiotic nodules in more detail, we analyzed transcriptomic data by Zorin et al. [29] on the transcript abundance in the root tips and symbiotic nodules at different stages of nodulation: 12 days, three weeks, and four weeks after rhizobial inoculation (dpi) (Figure 10). The transcripts of six *PsCEP* genes were found in this set of data: *PsCEP1*, *PsCEP2*, *PsCEP7*, *PsCEP13*, *PsCEP16*, and *PsCEP17*, where *PsCEP1*, *PsCEP2*, and *PsCEP7* were abundant at earlier stages of nodulation (12 dpi), and *PsCEP7*, *PsCEP13*, *PsCEP16*, and *PsCEP17* demonstrate high expression levels in mature nodules after four weeks of rhizobial inoculation.

**Figure 11:**
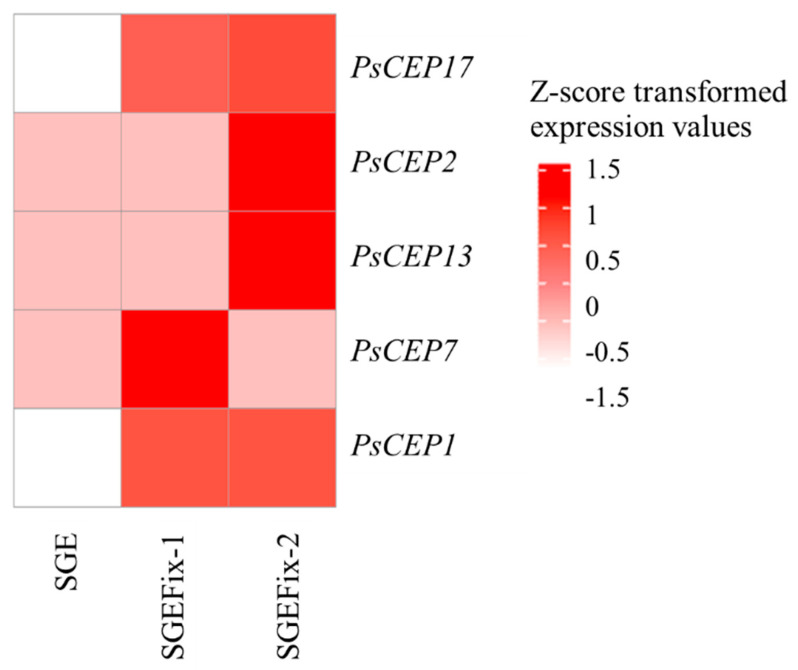
Expression levels of *PsCEP* genes in the nodules of symbiotic mutants defective in nitrogen fixation.

Moreover, we analyzed the transcriptomic data available for the pea mutants defective in nitrogen fixation, SGEFix^–^-1 (*Pssym40-1*) and SGEFix^–^-2 (*Pssym33-3*). These mutant lines carry mutations in transcription factor genes *PsEFD* and *PsIPD3*, respectively [30,31], which result in Fix^-^-phenotype (i.e., the mutant lines form white round-shaped ineffective nodules, where nitrogen fixation does not occur). Recently, the analysis of transcription changes in three-week-old nodules of SGEFix^–^-1 (*Pssym40-1*), SGEFix^–^-2 (*Pssym33-3*), and SGE (wild type) was performed [29]. Here, we analyzed the expression of *PsCEP* genes in mutant nodules with use of the available data (Figure 11). Interestingly, the *PsCEP1* and *PsCEP17* were among the genes differentially expressed between wild type (SGE) and both SGEFix^–^-1 and SGEFix^–^-2 mutant nodules. The nodules of Fix^–^- mutants do not fix nitrogen and, therefore, could experience nitrogen deficiency. Therefore, we could speculate that the upregulation of *PsCEP1* and *PsCEP17* in Fix^–^- mutants might be the consequence of nitrogen deficiency experienced by mutant plants and potentially indicative of ineffective nitrogen fixation. However, additional experiments are required to address this suggestion.

Next, we focused on six genes (*PsCEP1, PsCEP2, PsCEP7, PsCEP13, PsCEP16,* and *PsCEP17*) identified in transcriptomic data and studied their expression levels in the root and developing nodules by qPCR analysis.

### 2.6. Expression Analysis of the PsCEP Genes in Developing Nodules and in Response to Nitrate Treatment

**Figure 12:**
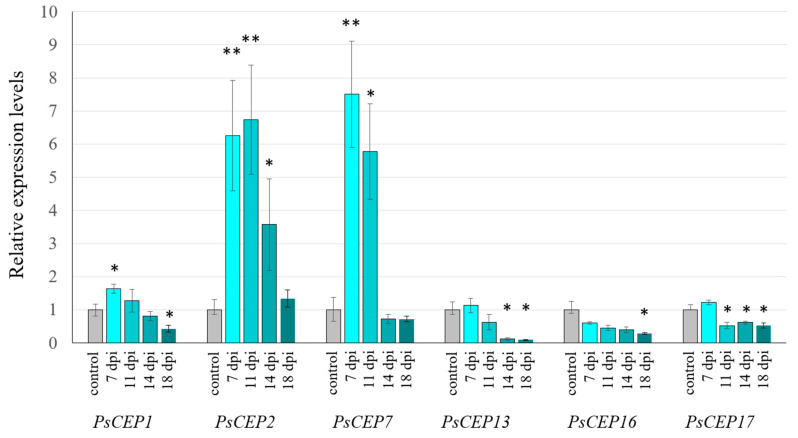
Expression levels of the *PsCEP* genes in developing nodules. “control” corresponds to non-inoculated roots, dpi–days post inoculation. Results are means ±SEM of 3-4 biological samples; Asterisks indicate statistically significant differences compared with the control (** *p* < 0.01; * *p* < 0.05) revealed by a Student’s *t* test. The gene expression levels were normalized to 1 against the expression found in the control roots.

To study the temporal expression patterns of the *PsCEPs* genes in developing nodules, we analyzed the expression levels of *PsCEP1, PsCEP2, PsCEP7, PsCEP13, PsCEP16,* and *PsCEP17* genes at different timepoints after inoculation (7, 11, 14, 18 dpi) by a qRT-PCR analysis. Among these genes, *PsCEP2* and *PsCEP7* genes demonstrated higher expression levels in developing nodules at 7–11 dpi in comparison with non-inoculated control roots (Figure 12), and at latter stages their expression levels were decreased. The *PsCEP1* gene also significantly increased the expression level in developing nodules at 7 dpi (*p* = 0.023, *t*-test). However, the difference between non-inoculated and inoculated roots was less pronounced for this gene (Figure 12). Therefore, based on the expression patterns in developing nodules, the *PsCEP1*, *PsCEP2,* and *PsCEP7* genes can be considered as the possible regulators of symbiotic nodule development.

Moreover, at later stages of nodule development, the *PsCEP1, PsCEP13, PsCEP16,* and *PsCEP17* genes demonstrated lower expression levels in comparison with non-inoculated roots. For example, at 18 dpi, the expression level of the *PsCEP1* gene was significantly lower in comparison with non-inoculated roots and the developing nodules at 7 dpi. This could be explained by the fact that, at later stages of nodule development, nitrogen fixation is initiated, and the expression levels of the *PsCEPs* genes decrease with the increasing nitrogen amount in nodulating roots.

**Figure 13:**
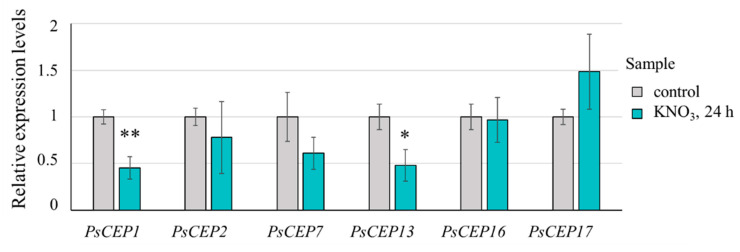
Expression levels of *PsCEP* genes in response to nitrate (10 mM KNO_3_, 24 h). Results are means ± SEM of 4–5 biological samples; Asterisks indicate statistically significant differences compared with the control (** *p* < 0.01; * *p* < 0.05) revealed by a Student’s *t* test. The gene expression levels were normalized to 1 against the expression level found in the control roots.

The *CEP* genes are known to change their expression levels depending on nutrient supply, especially nitrate, some of them being upregulated under low nitrate condition [2]. To study the possible effect of nitrate on PsCEP activity, we analyzed the expression levels of *PsCEPs* in response to nitrate treatment. Among six *PsCEP* genes analyzed by qPCR, the expression levels of *PsCEP1* and *PsCEP13* were significantly downregulated in response to nitrate addition (Figure 13). Therefore, these two genes could be involved in the regulation of nitrate-dependent processes in plants.

Furthermore, to analyze the possible effect of nitrate treatment on the activity of the *PsCEPs* genes during nodulation, we assessed the expression levels of *PsCEPs* in nodulated roots of plants treated with nitrate (15 mM KNO_3_) and control plants with no nitrate treatment four weeks post inoculation with rhizobia. Nitrate is known to inhibit nodulation and nitrogen fixation in legume plants [13,32]. Accordingly, in our experiment, nitrate-treated plants had reduced nodule number (see figure legend to Supplementary Figure S3) in comparison to the control plants. Interestingly, the expression levels of the *PsCEP2* and *PsCEP13* genes were significantly increased in the nodulated roots of nitrate-treated plants in comparison to the control group with no nitrate addition according to qRT-PCR analysis (Supplementary Figure S3). The mean expression levels of the *PsCEP7* gene were also higher in nitrate-treated nodulated roots. However, this increase was not statistically significant (*p* = 0.051, Student’s *t* test). Interestingly, the *PsCEP2* and *PsCEP13* genes were among the DEGs upregulated in ineffective SGEFix^–^-2 mutant nodules in comparison to the wild type nodules. Moreover, a significant decrease in *PsCEP* (including *PsCEP13*) expression at later stages of nodulation, when a nitrogen fixation is initiated, is in line with our observation that the impaired nitrogen fixation is accompanied by an increase in *PsCEP*s expression. The increased expression of the *PsCEP* genes in the nodulated roots of nitrate-treated plants could be explained by the reduced number of nodules and less efficient nitrogen fixation in comparison to the control roots, which, in turn, could result in a lower amount of nitrogen (in a form of ammonia) in the roots of the treated group in comparison to the control one. However, additional experiments are required to check this suggestion.

## 3. Discussion

In this study, we identified 21 genes encoding CEP peptides in *P. sativum*. According to phylogenetic analysis, the CEP domains found in PsCEP proteins were clustered into two subgroups, CEP-I and CEP-II. The group I CEP peptides demonstrate higher conservation within the CEP domain with the conserved Pro4, Pro7, and Pro11 residues, while CEP-II peptides show significant divergence in the N-terminal part with highly conserved C-terminal part of the CEP domain with the Pro9 and Pro11 residues within PSPG sequence. This is consistent with the observation made by Delay et al., who also divided CEP peptides found in angiosperm into two subgroups based on the similar principle with the same conserved Pro residues [2]. Previously, the Pro11 residue was shown to be hydroxylated and triarabinosylated in the MtCEP1 peptide and this modification could be important for CEP peptide activity [4]. The Pro4 and Pro7 residues conserved in the CEP-I were found to be hydroxylated in the MtCEP1 peptide [4], suggesting the importance of these positions for CEP peptide activity. The Pro residues in these positions were absent in the CEP-II subgroup. Since the Pro residues in posttranslationally modified peptides are potential sites of hydroxylation and triarabinosylation, which, in turn, is important for their function and receptor recognition, we could speculate that CEP-I and CEP-II peptides may have different receptor-binding specificity and/or activity.

For most of *PsCEP* genes, the putative orthologues from *M. truncatula* were identified, which was supported by the phylogenetic and microsynteny analysis. For the *MtCEP15* gene, three putative pea orthologues were found with high sequence similarity, suggesting the recent duplication events. The homologous pairs of MtCEP and PsCEP proteins have the same number of CEP domains, except for the CEP6 and CEP9 proteins. In pea, these proteins have only one CEP domain, while *M. truncatula* MtCEP6 and MtCEP9 have two CEP domains. Two multidomain PsCEP proteins containing two distinct CEP-I domains were identified in pea: PsCEP1 and PsCEP7. Interestingly, the closest homologues of both these genes were shown to be positive regulators of nodule morphogenesis in *M. truncatula* [3,11]. Moreover, the PsCEP22 and PsCEP24 proteins contain two CEP-II domains. At the same time, the CEP-I domain and the CEP-II domain never occurred in the same precursor protein. Finally, the PsCEP20, PsCEP22, and PsCEP23 proteins appeared to have conserved PIP-like domains in addition to the CEP domains. In *A. thaliana*, the expression of the *PIP* genes was induced in response to a variety of pathogens and elicitors, and their overexpression resulted in enhanced immune responses and pathogen resistance, suggesting the role of PIP peptides in the modulation of plant immunity [33]. It is of great interest to explore the role of PIP-like domains found in multidomain PsCEP precursor proteins.

The analysis of transcriptomic data revealed six *PsCEP* genes (*PsCEP1*, *PsCEP2*, *PsCEP7*, *PsCEP13*, *PsCEP16*, and *PsCEP17*) with relatively high expression levels in the root and symbiotic nodules. Interestingly, the *PsCEP1* and *PsCEP17* genes were among the genes differentially expressed between wild type nodules and nodules of the mutants with impaired nitrogen fixation (Fix^–^-mutants SGEFix^–^-1 (*Pssym40-1*) and SGEFix^–^-2 (*Pssym33-3*). Therefore, we could suggest that the upregulation of these genes in symbiotic nodules could be a marker of ineffective nitrogen fixation; however, additional data are required to check this hypothesis.

The expression analysis of the *PsCEP* genes during nodulation revealed that the *PsCEP2* and *PsCEP7* genes were highly upregulated in developing nodules. Moreover, a less pronounced induction of the *PsCEP1* gene was observed, suggesting the role of these genes in symbiotic nodule development in pea. In *M. truncatula*, the homologues of these genes, *MtCEP1* and *MtCEP7,* were characterized as the positive regulators of symbiosis [3,11]. Moreover, *MtCEP7* was found to be activated by the NIN transcription factor–a master transcriptional regulator of symbiosis [11]. The *PsCEP7* gene could be also a target of the NIN transcription factor, since we have identified two putative NBS motifs in its promoter based on the consensus motif described by Laffont et al. [11]). Moreover, one NBS was found in the *PsCEP1* gene, suggesting that its upregulation in developing nodules could be also mediated by NIN. However, additional experiments are required to check this suggestion. Recently, the MtCEP2 peptide was shown to promote nodulation in *M. truncatula* and inhibit lateral root formation [12]. Moreover, *MtCEP2* was confirmed to control lateral root number and nodulation redundantly with *MtCEP1* and *MtCEP12* based on the CRISPR/Cas9-based multigene editing approach [12]. Based on these observations, we could suggest that *PsCEP1, PsCEP2,* and *PsCEP7* genes could be the key candidates for the regulators of symbiotic nodule development in pea.

Finally, we studied the effect of nitrate treatment on the expression levels of *PsCEP* genes and found that *PsCEP1* and *PsCEP13* were significantly downregulated in response to nitrate addition. In *M. truncatula,* the MtNLP1 transcription factor was recently shown to bind to the *MtCEP1* promoter through specific half NRE (hNRE) motifs to repress its expression in the presence of nitrate [15]. The nNRE motif was also found in the *PsCEP1* gene promoter, suggesting that it could be targeted by an NLP transcription factor in pea. However, no putative hNRE was found in *PsCEP13.* The nitrate-mediated regulation of the *PsCEP1* gene is consistent with its downregulation in mature nodules at 18 dpi, when nitrogen fixation is initiated and the nitrogen amount increases, and the upregulation of *PsCEP1* in the symbiotic nodules of Fix^–^- mutants experiencing nitrogen deficiency. Therefore, the *PsCEP1* gene, which is regulated by both nitrate and rhizobia signaling pathways and has both NBS and NRE elements in its promoter, could be a target of nitrate-dependent regulation of nodulation in pea. Future experiments, including a functional characterization of the *PsCEP1* gene, should be performed to elucidate its role in symbiotic nodule development and nitrogen response in pea.

## 4. Materials and Methods

### 4.1. Identification of the PsCEP Genes and Their Phylogenetic Analysis

To identify genes encoding CEP peptides in *P. sativum,* BLASTn [34] analyses with *MtCEP* genes as queries were used against genomic (https://urgi.versailles.inra.fr/download/pea/, accessed on 5 July 2022) [20] and transcriptomic data [21] of pea cv. Cameor. Sequences of *MtCEP* genes were obtained from The *Medicago truncatula* Small Secreted Peptide Database [19]. The analysis of the exon-intron structure of the genes was carried out by aligning the full-length and coding sequences of the corresponding *PsCEP* genes. EMBOSS Transeq was used to translate nucleic acid sequence to corresponding peptide sequences [35]. Phylogenetic analysis was performed using the MEGAX program (https://www.megasoftware.net/, accessed on 11 July 2022). Nucleotide sequences were aligned with the muscle algorithm, and the phylogenetic tree was generated using the maximum likelihood method with 1000 bootstrap replicates.

### 4.2. Chromosome Localization of the PsCEP Genes and Microsynteny Analysis

Chromosome localization of the *PsCEP* genes was performed with the MG2C online software with default settings (http://mg2c.iask.in/mg2c_v2.0/, accessed on 11 July 2022). Microsynteny analysis of *PsCEP* and *MtCEP* genes was performed using the tblastn tool from NCBI C++ toolkit (version 2.9.0+). Visualization of gene clusters on *P. sativum* and *M. truncatula* chromosomes was carried out using an R script in the R programming language and packages ggplot2 (H. Wickham. ggplot2: Elegant Graphics for Data Analysis. Springer-Verlag New York, 2016.) and gggenes (https://github.com/wilkox/gggenes, accessed on 11 July 2022).

HSDFinder [22] and MCScanX [23] were used for analysis of gene duplication and pairwise collinear relationships of the *PsCEP* genes.

### 4.3. Promoter Analysis of the PsCEP Genes

The 2-kb sequences upstream of start-codon were used for the promoter analysis. PlantRegMap platform [24] was used to search conserved *cis*-regulatory with *p*-value 1e-4 as a threshold. Putative NBS and NRE motifs were identified using MAST [36] and FIMO [37] algorithms available on the MEME suite server (https://meme-suite.org/meme/, accessed on 8 July 2022).

### 4.4. The Structural Characterization of the PsCEP Proteins and Prediction of Physicochemical Properties of CEP Peptides

Signal IP ver.6.0 (https://services.healthtech.dtu.dk/service.php?SignalP, accessed on 8 July 2022) was used for N terminal signal peptide prediction of PsCEP proteins. The CEP domains were identified using MEME tool (https://meme-suite.org/meme/, accessed on 8 July 2022), FIMO tool [37] and manual search. Sequence logos were created using the WebLogo online service (https://weblogo.berkeley.edu/logo.cgi, accessed on 11 July 2022).

The sequences of mature CEP peptides without signal peptide were analyzed using the Antimicrobial Peptide Database [38] to predict its physicochemical properties. The isoelectric point of each peptide was predicted using the IPC 2.0 tool [39].

### 4.5. Expression Analysis of PsCEP Genes by a qPCR Analysis

#### 4.5.1. Plant Growth Conditions

*P. sativum* cv. Frisson seeds were surface sterilized with concentrated sulphuric acid for 8 min, then washed several times with sterile water and transferred to 1% water agar plates for germination (3–4 days in the dark at room temperature). For the nodulation experiment, plants were grown in aeroponics system containing aeroponic nutrient medium [40]. Plants were inoculated with *Rhizobium leguminosarum* bv. *viciae* RCAM1026 (ARRIAM, WDCM 966) culture grown on YMB (Yeast Extract Mannitol) plates (diluted to the final concentration OD_600_ = 0.02 − 0.05). Non-inoculated control roots together with the inoculated roots were used for RNA extraction.

For the nitrate treatment experiment, pea plants were grown in a hydroponic system filled with nitrogen-free aeroponic nutrient medium [40]. KNO_3_ was added to the medium to a final concentration of 10 mM, and pea roots were harvested in the 24 h after that. Untreated plants were used as a control.

For the experiment with nitrate treatment of rhizobia-inoculated plants, pea seeds (“Nemchinovsky” cultivar) were surface sterilized with concentrated sulphuric acid for 8 min, washed several times with sterile water, and then transferred to 1% water agar plates for germination (3–4 days in the dark at room temperature). After that, the seedlings were transferred to 2 L metal pots filled with sterilized quartz sand and inoculated with *Rh. leguminosarum* bv viciae strain RCAM1026 upon planting. Plants were supplied with mineral nutrition solution as described in Sulima et al., 2019 [41]. For nitrate treatment, KNO_3_ was added to the mineral nutrition solution to the concentration of 15 mM. Plants were watered as needed with distilled water, and once a week were supplied with mineral nutrition solution (with or without KNO_3_), 150 mL per pot.

#### 4.5.2. RNA Extraction and qPCR Analysis

RNA was extracted from the roots of individual plants using TRIZol reagent according to the manufacturer’s instructions (Thermo Scientific, Waltham, MA, USA). Rapid Out DNA Removal Kit was used for DNase treatment (Thermo Scientific, Waltham, MA, USA). RNA concentration and quality were analyzed using NanoDrop 2000c UV-Vis Spectrophotometer (Thermo Scientific, Waltham, MA, USA). qRT-PCR analysis was performed using the C1000 thermal cycler with CFX-96 real-time PCR detection system (Bio-Rad Laboratories, Alfred Nobel Drive, Hercules, CA, USA) with SYBR Green intercalating dye (Sintol, Moscow, Russia). The data were analyzed by the CFX Manager software (Bio-Rad Laboratories, Alfred Nobel Drive Hercules, CA, USA) with the 2^−ΔΔCt^ method with ubiquitin and tubulin genes used as the reference genes. Primers were designed using Primer3 Plus software (https://www.primer3plus.com/, accessed on 11 July 2022). The qRT-PCR reactions were run in three technical repeats. Primers used for qRT-PCR are listed in Table S4. The specificity of PCR amplification was confirmed with dissociation curves (55–95 °C).

Student’s *t*-test was used to compare gene expression levels in the experiment with nitrate treatment, four biological replicates were used for each condition. To study gene expression during nodulation, three biological experiments were performed, where the root segments from three plants were combined in one sample for each timepoints. The results of one representative experiment are shown.

### 4.6. Expression Analysis of PsCEP Genes Using Transcriptomic Data

Transcriptomic analysis was performed using the reads obtained by Zorin et al. [29]. All raw reads were processed to filter out adaptor sequences and low-quality sequences using BBDuk from BBMap toolkit (https://jgi.doe.gov/data-and-tools/software-tools/bbtools/, accessed on 11 July 2022). High quality reads were mapped to the reference *P. sativum* cv. Cameor genome assembly using STAR (ver. 2.7.6a) [42]. The quality of the data and the distribution of replicates were evaluated by the principal component analysis. Differential expression analysis was conducted using DESeq2 (ver. 1.34.0) package [43] in R programming environment (ver. 4.1.2). The differentially expressed genes were considered to be significant at the level of the adjusted *p*-value of < 0.05. All heatmaps were built on the basis of a 1-Pearson correlation matrix calculated on normalized per million and logarithmic (log2) expression values transformed into a z-score using edgeR (ver. 3.20.9) [44] and pheatmap package in R.

## 5. Conclusions

The present study provides a comprehensive analysis of the *CEP* genes in pea genome. Interestingly, three genes were identified containing the conserved motifs corresponding to the PIP peptide consensus sequences in addition to the CEP domains. This finding suggests that these two groups of post-translationally modified peptides have a common evolutionary origin, blurring the lines between these peptide families. Moreover, the analysis of transcriptomic data and expression analysis by qPCR revealed candidate regulators of symbiotic nodulation in pea, including the *PsCEP1, PsCEP7,* and *PsCEP2*, which should be investigated in further functional studies.

## Data Availability

Not applicable.

## Supplementary Materials

The following supporting information can be downloaded at: https://www.mdpi.com/article/10.3390/ijms232314875/s1, Supplementary Figure S1: The amino acid sequences of the MtPIP8 protein. The CEP and PIP-like domains are highlighted. Supplementary Figure S2. Putative NIN/NLP and NIN-specific binding sites found in the promoters of the *PsCEP* genes using the motifs described by Nishida et al., 2021 [25]. Asterisks of the same color mark similar cis-regulatory elements. Supplementary Figure S3. Expression levels of the *PsCEP* genes in mature nodules (pea cv. Nemchinovsky 4 weeks after inoculation with rhizobia). Control plants (“no nitrate”) were not treated with nitrate, “15 mM KNO_3_” corresponds to the experimental group treated with 15 mM KNO_3_ roots once a week. Results are means ± SEM of 4 biological samples. Asterisks indicate statistically significant differences compared with the control group (* *p* < 0.05) revealed by a Student’s *t* test. The gene expression levels were normalized to 1 against the expression found in the control group (“no nitrate”). The nodule numbers were as follows: “no nitrate”: 125.60 ± 3.79 (mean ± SE); *n* = 30; “15 mM KNO_3_”: 81.03 ± 3.08 (mean ± SE); *n* = 30, (*p* = <0.001, *t*-test). Table S1: The characteristics of *PsCEP* genes identified in pea genome. Table S2: Conserved domains identified in the PsCEP proteins. Table S3: Signal peptides identified in the PsCEP proteins. Table S4: Primers used for qPCR.
