# Supplementary material for: Identification and Expression Analysis of the C-TERMINALLY ENCODED PEPTIDE Family in Pisum sativum L
Source: Int J Mol Sci. 2022 Nov 28;23(23):14875. doi: 10.3390/ijms232314875 (PMC9739355; doi:10.3390/ijms232314875)
Supplement: Supplementary file 1 [file ijms-23-14875-s001.zip › Supplementary_Figures_updated_27_11.pdf]

Supplementary Figure S1

MtPIP8

CEP PIP-like

MDTRLKSFVTFLTFILLVSLFVSLLPNVSEARPFLSPLQGREGVIGEVNGV**FRTLKDAGPSPGVGH**KLKNLQKLHNFEDHKLNTVQG**LGVIKHSGPSPGEGH**KYITNNNNS

Supplementary Figure S1. The amino acid sequences of the MtPIP8 protein. The CEP and PIP-like domains are highlighted.

Supplementary Figure S2

A NIN/NLP-binding sites

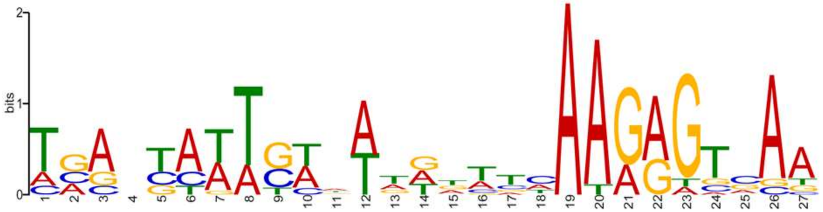

PsCEP4.1 *	-650	T G A T G A T T C A A A A A A A A A A A G A G A C A A	-677
PsCEP4.2	-441	T G A T T C A T G T T T G T T T T A A A A A G G A A A	-468
PsCEP4.3	-45	A G C T C A A T G C A A T G A A G C A A G G G T G A G	-72
PsCEP6	-1509	T G A A T A T T G T G A C T C A A G A A G A T T C A A	-1536
PsCEP9.1 *	-1795	A G G A T C A T G C T T T G G T C A A A G A G T C A T	-1768
PsCEP9.2	-872	T C A C T C G A G T G A A G A G T C A A G A G C A A G	-899
PsCEP12.1	-209	T C A A G A T T C T C T T T T T G T A A A G G T C A A	-236
PsCEP12.2	-99	T A G G C A T T G A G A G A G T T C A A A A G T T G A	-126
PsCEP14 *	-226	T A A T T A T T C A A T G A A T T C A A A A G T A A C	-253
PsCEP15b	-520	A C A C T A A T G T T T T G G C C T A A G G G G C C A	-493
PsCEP22.1	-1007	C A G G C A A A C A A A A A T T T C A A G A G T G A A	-1034
PsCEP22.2	-606	T C A A G T A T T A C A T A T T T T A A G G G C C A A	-633
PsCEP23.1	-1418	T G C C C T T A C T A A A G G C C C A T G G A T G A T	-1445
PsCEP23.2	-304	C A A C T A T A T A T T T T T A T A A A G A G T G A A	-277

B NIN-specific binding sites

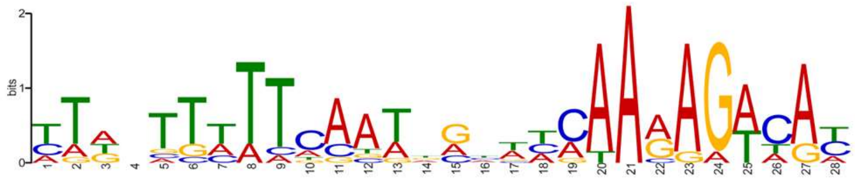

PsCEP4 *	-649	C T G A T G A T T C A A A A A A A A A A A G A G A C A A	-677
PsCEP6	-1700	C T A G T T C A T C G A T G G T A C C T A A A G A T G T	-1672
PsCEP7	-334	A T T T G T T T T T A A G T A C A C C A A A G G A C A T	-306
PsCEP9.1 *	-1796	T A G G A T C A T G C T T T G G T C A A A G A G T C A T	-1768
PsCEP9.2	-864	T G G T T G A T T C A C T C G A G T G A A G A G T C A A	-892
PsCEP9.3	-472	G T G T T T G T T G A T A C T A T T G A A A A G G T A A	-500
PsCEP13	-1109	C T T C T T T T C C A A A A G C C T C A A G A G A A A C	-1081
PsCEP14 *	-225	T T A A T T A T T C A A T G A A T T C A A A A G T A A C	-253
PsCEP20	-1543	T T A T T C T T A A C A A T C C T A A A A A A G T T G T	-1571
PsCEP23	-624	A A T A T T T T T A A G T G C T T A C A A A A G A C A C	-596

Supplementary Figure S2. Putative NIN/NLP and NIN-specific binding sites found in the promoters of the *PsCEP* genes using the motifs described by Nishida et al., 2021 [25]. Asterisks of the same color mark similar *cis*-regulatory elements

Supplementary Figure S3

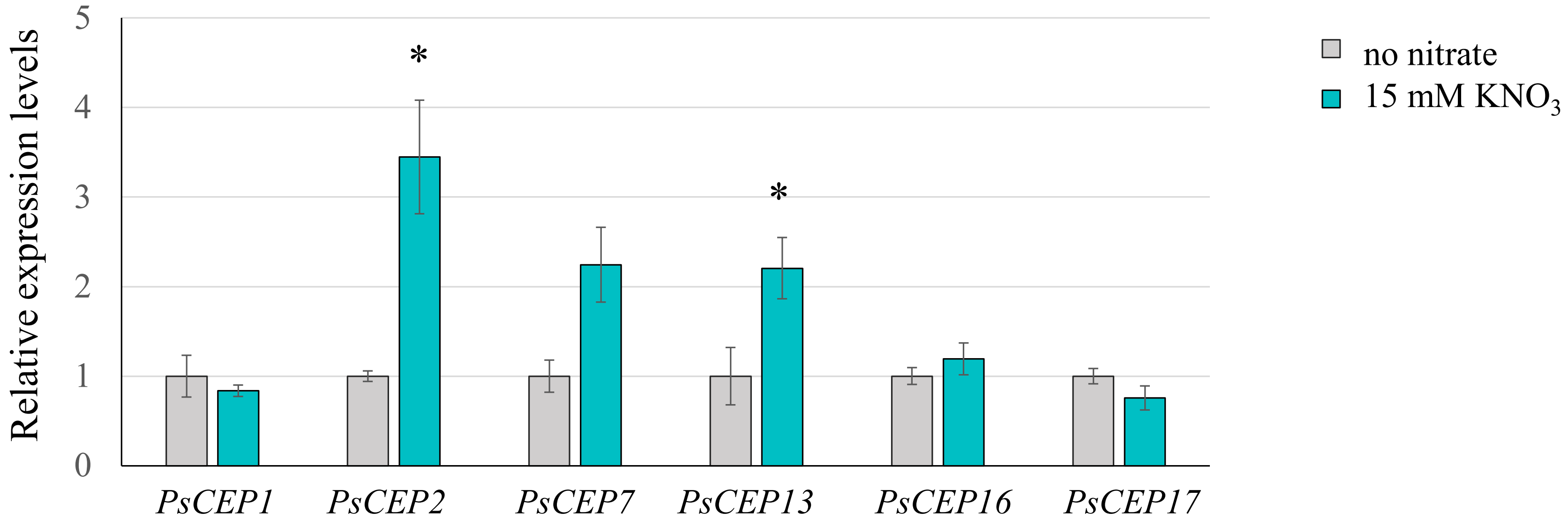

Supplementary Figure S3. Expression levels of the *PsCEP* genes in mature nodules (pea cv. Nemchinovsky 4 weeks after inoculation with rhizobia). Control plants ("no nitrate") were not treated with nitrate, "15 mM KNO_3" corresponds to the experimental group treated with 15 mM KNO_3 roots once a week. Results are means ±SEM of 4 biological samples. Asterisks indicate statistically significant differences compared with the control group (* $p < 0.05$) revealed by a Student's t test. The gene expression levels were normalized to 1 against the expression found in the control group ("no nitrate"). The nodule numbers were as follows: "no nitrate": 125.60 ± 3.79 (mean ± SE); n=30; "15 mM KNO_3": 81.03 ± 3.08 (mean ± SE); n=30, (P = <0.001, t-test)
